# Mapping potentially avoidable premature mortality in Mexico: subnational, sex, and age group trends

**DOI:** 10.1590/0102-311XEN178723

**Published:** 2024-08-26

**Authors:** Andrés Castañeda Prado, Iliana Yaschine Arroyo, Guillermo Salinas-Escudero, Juan Pablo Gutiérrez

**Affiliations:** 1 Facultad de Medicina, Universidad Nacional Autónoma de México, Cuidad de México, México.; 2 Programa de Maestría y Doctorado en Ciencias Médicas, Odontológicas y de la Salud, Universidad Nacional Autónoma de México, Cuidad de México, México.; 3 Programa Universitario de Estudios del Desarrollo, Universidad Nacional Autónoma de México, Cuidad de México, México.; 4 Centro de Estudios Económicos y Sociales en Salud, Hospital Infantil de México Federico Gómez, Cuidad de México, México.

**Keywords:** Premature Mortality, Healthcare Disparities, Socioeconomic Factors, Mortalidad Prematura, Disparidades en Atención de Salud, Factores Socioeconómicos, Mortalidade Prematura, Disparidades em Assistência à Saúde, Fatores Socioeconômicos

## Abstract

This study aimed to analyze the trends and disparities in preventable or treatable mortality rates among different age groups, sexes, and states in Mexico from 2000 to 2019. Using national data from 2000 to 2019, we examined potentially avoidable premature mortality (PAPM) rates, disaggregated into preventable and treatable deaths. Trends over time were visualized using the average annual percent change (AAPC) derived from joinpoint analysis. Subnational analysis was conducted to identify state-specific trends for each sex and age group. The national PAPM rate decreased from 297 deaths per 100,000 in 2000 to 281 per 100,000 in 2019. Potentially preventable premature mortality (PPPM) rates were more pronounced than potentially treatable premature mortality (PTPM) rates, with 170 deaths per 100,000 and 111 per 100,000, respectively. Sex-based disparities were observed particularly in the working-age population. Our analysis at the state level revealed significant differences in trends, as certain regions experienced reductions while others rises. These disparities became more evident when examining the different aspects of PAPM, especially in terms of PTPM. Our study highlights the differences in PAPM rates across age groups, sexes, and states in Mexico. Despite a general downward trend, upward trends were observed in the male working-age group. There was also wide variation among states, highlighting the need to use PAPM in conjunction with other health metrics for a holistic health analysis.

## Resumen

Este estudio tuvo como objetivo analizar las tendencias y disparidades en las tasas de mortalidad evitable o tratable en diferentes grupos de edad, sexo y estados de México en el período de 2000 a 2019. Con base en datos nacionales de 2000 a 2019, se analizaron las tasas de mortalidad prematura potencialmente evitable (MPPE), dividiéndolas en muertes evitables y tratables. Las tendencias a largo plazo se observaron mediante el cambio porcentual promedio anual (CPPA) obtenido del análisis de regresión *joinpoint*. Se realizó un análisis subnacional para identificar las tendencias específicas de cada estado por sexo y grupo de edad. La tasa nacional de MPPE disminuyó de 297 muertes por cada 100.000 en el año 2000 a 281 por cada 100.000 en el 2019. Las tasas de mortalidad prematura potencialmente prevenible (MPPP) fueron mayores que las de mortalidad prematura potencialmente tratable (MPPT), con 170 muertes por cada 100.000 y 111 por cada 100.000, respectivamente. Este análisis reveló variaciones sustanciales en las tendencias por estado, ya que algunas regiones tuvieron disminución mientras que otras un aumento. Estas disparidades se hicieron más evidentes cuando los aspectos de la MPPE se analizaron con más detalle, especialmente en términos de MPPT. Este estudio destaca las variaciones en las tasas de la MPPE entre grupo de edad, sexo y estados en México. A pesar de una tendencia general hacia el declive, se observaron tendencias al alza en la población masculina en edad de trabajar. Además, se observaron considerables variaciones entre estados, lo que muestra la necesidad de aplicar la MPPE en conjunto con otros indicadores de salud para realizar un análisis holístico de salud.

## Resumo

Este estudo teve como objetivo analisar as tendências e disparidades nas taxas de mortalidade evitável ou tratável em diferentes faixas etárias, sexos e estados do México de 2000 a 2019. Usando dados nacionais de 2000 a 2019, examinamos as taxas de mortalidade prematura potencialmente evitável (MPPE), dividindo-as entre mortes evitáveis e tratáveis. As tendências ao longo do tempo foram visualizadas usando a variação percentual média anual (VPMA) obtida a partir da análise de regressão *joinpoint*. Foi efectuada uma análise subnacional para identificar as tendências específicas de cada estado para cada sexo e faixa etária. A taxa nacional de MPPE diminuiu de 297 mortes a cada 100 mil em 2000 para 281 a cada 100 mil em 2019. As taxas de mortalidade prematura potencialmente prevenível (MPPP) foram mais acentuadas do que as mortalidade prematura potencialmente tratável (MPPT), com 170 mortes a cada 100 mil e 111 a cada 100 mil, respetivamente. A nossa análise a nível estatal revelou variações substanciais nas tendências, uma vez que certas regiões registaram reduções enquanto outras apresentaram aumentos. Essas disparidades tornaram-se mais evidentes quando os aspectos da MPPE foram analisados mais detalhadamente, especialmente em termos de MPPT. Nosso estudo destaca as variações nas taxas de MPPE entre faixas etárias, sexos e estados no México. Apesar de uma tendência geral para o declínio, foram observadas tendências de aumento na população masculina em idade ativa. Além disso, foram observadas variações consideráveis entre os estados, o que reforça a necessidade de aplicar a MPPE em conjunto com outros indicadores de saúde para efetuar uma análise holística da saúde.

## Introduction

Data from 2019 collected by the Organisation for Economic Co-Operation and Development (OECD) indicated that over 3 million people aged under 75 years died prematurely in OECD countries, accounting for more than 25% of all deaths. Of these, approximately 1.9 million deaths could have been avoided by implementing effective primary prevention and public health initiatives, while more than 1 million could have been mitigated with timely and efficient healthcare interventions. Notably, Mexico had one of the highest rates, surpassing the average OECD rate ^1^.

Potentially avoidable premature mortality (PAPM) is an indicator used by the Pan American Health Organization (PAHO) and the OECD, derived from the concept of “avoidable deaths” ^2^ ^,^ ^3^. PAPM is divided into two subcategories: potentially preventable premature mortality (PPPM) and potentially treatable premature mortality (PTPM). PPPM encompasses deaths that could be averted with primary prevention and public healthcare measures, while PTPM refers to deaths that could be delayed or avoided with prompt, high-quality medical care. This framework is based on the belief that numerous deaths can be prevented either by disease prevention (reducing incidence) or by effective management or treatment post-onset (reducing lethality), which makes PAPM particularly relevant to support public policy actions ^4^ ^,^ ^5^. PAPM covers deaths that can be prevented with effective public health interventions, such as vaccination and smoking cessation, and deaths that are treatable with efficient medical care, such as those caused by sepsis, breast cancer, and asthma ^6^. PAPM also includes deaths in people under 75 that could be avoided with appropriate living conditions and access to healthcare.

Avoidable mortality serves as an indirect measure to evaluate changes in mortality associated with public health policies, assuming that the trend should be downward, with the ultimate goal of eliminating all instances of PAPM ^7^.

The analysis of PAPM at the subnational level reveals regional disparities, thereby guiding specialized interventions. A localized focus, complemented by the evaluation of trends across various age groups and sexes, promotes efficient resource allocation and enhances targeted population health outcomes ^8^ ^,^ ^9^.

In Mexico, assessing preventive ambulatory primary care performance by region, sex, and age is crucial to understand regional disparities and the impact of socioeconomic factors on health. This focused analysis enables policymakers to identify specific vulnerabilities and design interventions tailored to the unique needs and challenges of different demographic groups.

Since 2003, the health reform known as Popular Isurance (*Seguro Popular*) has given each state autonomy to manage its own healthcare system. This autonomy may contribute to variations in PAPM, particularly affecting differences in PTPM ^10^ ^,^ ^11^. Given the diversity of the population and the different socioeconomic conditions across states, these factors result in unequal health outcomes ^12^ ^,^ ^13^ ^,^ ^14^ ^,^ ^15^.

For example, southern states consistently show poorer performances in health indicators such as child stunting, under-five mortality, and neonatal mortality ^14^. This disparity is further underscored by the higher infant mortality rate in areas with higher poverty, where 22.14 more deaths occur per 1,000 live births compared to areas with lower poverty ^13^. These findings highlight the need for localized assessments and targeted interventions to address health disparities and improve overall well-being in Mexico’s complex healthcare landscape.

PAPM outcomes vary due to the combined influence of sex, age, and geography, all of which interact with factors such as biological differences, lifestyles, socioeconomic conditions, and access to healthcare ^16^. For instance, inherent biological differences between sexes can affect disease susceptibility and treatment response, leading to different outcomes ^7^ ^,^ ^17^. In OECD nations, preventable mortality rates were 2.5 times higher in men than in women ^3^. Age is another critical factor; the causes of preventable and treatable mortality differ among working-age populations, older adults, children, and adolescents, reflecting the prevalence of specific diseases and conditions within each group. For example, 98% of noncommunicable disease-related deaths in 2019 occurred in individuals aged 15 years and older ^18^, unlike vaccine-preventable diseases and other illnesses that prevail in early childhood.

In the Mexican context, unique geographic phenomena, such as the variation in interregional levels of violence, directly and indirectly influence PAPM rates by affecting access to healthcare, socioeconomic conditions, and lifestyles ^19^ ^,^ ^20^. Additionally, population characteristics and local health policies differ among Mexican states ^21^.

Recognizing the complex interplay of factors that contribute to PAPM is essential for developing targeted public health strategies to address health disparities in Mexico. Understanding these factors enables policymakers and healthcare providers to identify vulnerable populations and implement tailored interventions, thereby fostering health equity and ensuring access to quality healthcare services.

This study aims to describe the trends and disparities in preventable and avoidable mortality rates from 2000 to 2019 at the subnational (state) level, segmented by sex and four age groups: preschool age (0-4 years), school age (5-14 years), working age (15-64 years), and post-working age (65-74 years). The working-age group was defined as 15-64 years, as established by the OECD ^22^.

## Methods

An ecological multigroup study of death records in Mexico from 2000 to 2019 was designed to examine trends in PAPM rates. The period of analysis extends to 2019, the pre-COVID-19 era. The onset of the global pandemic in 2020 dramatically disrupted healthcare systems worldwide, including Mexico, and could have severely skewed the data, which makes it essential to limit the analysis to the pre-pandemic period. The methodology is articulated in two main phases: (a) computation of the PAPM rate using statistical software and standardization methods; (b) analysis of trends in PAPM and its components using Joinpoint regression analysis, version 5 (https://surveillance.cancer.gov/joinpoint/) at the national and subnational levels. These phases were instrumental in providing detailed insights into mortality patterns and the effectiveness of public health interventions.

First, the PAPM rate was computed using R version 4.2.1 (http://www.r-project.org), based on state population projections from the Mexican Government ^23^. The PAPM rate encompassed the sum of deaths from 2000 to 2019 considered preventable or treatable, sourced from deaths registered by the Mexican National Institute of Statistics and Geography (INEGI; acronym in Spanish) ^24^. The causes reported by the INEGI were classified according to the OECD/EUROSTAT list, last updated in January 2022 ^25^. The direct method of standardization was applied, dividing the population into five-year age groups in accordance with the World Health Organization (WHO) standard population classification for the period 2000-2025 ^26^. To compute the PAPM rate, the number of potentially avoidable premature deaths was divided by the total population at the midpoint of the period and then the result was multiplied by 100,000. Rates by subperiod (2000-2004, 2005-2009, 20102014, and 2015-2019) were calculated using the population at the midpoint of the period.

Joinpoint regression analysis was then conducted to assess trends in PAPM from 2000 to 2019. This approach helped to identify inflection points and calculate average annual percentage changes (AAPCs) via log-linear function of age-standardized rates. The AAPC served as a composite measure for assessing trends over specific intervals. Segmented regression analysis was used to estimate the annual percentage change (APC) between breakpoints, assessing the goodness of fit with Bayesian information criteria and degrees of freedom ^25^. The analysis used the joinpoint regression program developed by the Statistical Research and Applications Branch of the National Cancer Institute, configured to detect up to three joinpoints. AAPCs and 95% uncertainty intervals (95%UI) for 2000-2019 and subperiods (2000-2004, 2005-2009, 2010-2014, and 2015-2019) were calculated by sex (male, female) and by age group: preschool age (0-4 years); school age (5-14 years); working age (15-64 years); and post-working age (65-74 years). To do this, the joinpoint regression program uses the following segmented regression model to estimate the annual percentage change between breakpoints:

$$\log\left[\left(y\right)=\beta 0+\beta 1*x\right]$$

In which: *y* is the rate for a given year; *x* is the time variable (the year); *β0* is the intercept of the mode; and *β1* is the coefficient representing the slope of the trend. To calculate the APC, the program extracts the slopes from the model and then converts them to a percentage change per year. The APC equation is:

$$\text{APC=}\left(e^{\left(\beta \right)}-1\right)*\text{100}$$

In which *β* is the slope of the respective segment.

Lastly, to compute the AAPC, the following model was used:

$$\text{AAPC=}\left\{\text{exp}\left(\frac{\sum w_{i}b_{i}}{\sum w_{i}}\right)-1\right\}*\text{100}$$

In which *b*_*i*_ is slope coefficient for the *i* ^th^ segment, with *i* indexing the segments in the desired range of years, and *w*_*i*_ is the length of each segment in the range of years.

The trends for each federal entity were analyzed in the different rate types (PAPM, PPPM, and PTPM) and sex. This process was done for each age group for the period from 2000 to 2019.

## Results

### National analysis

**Figure 1:**
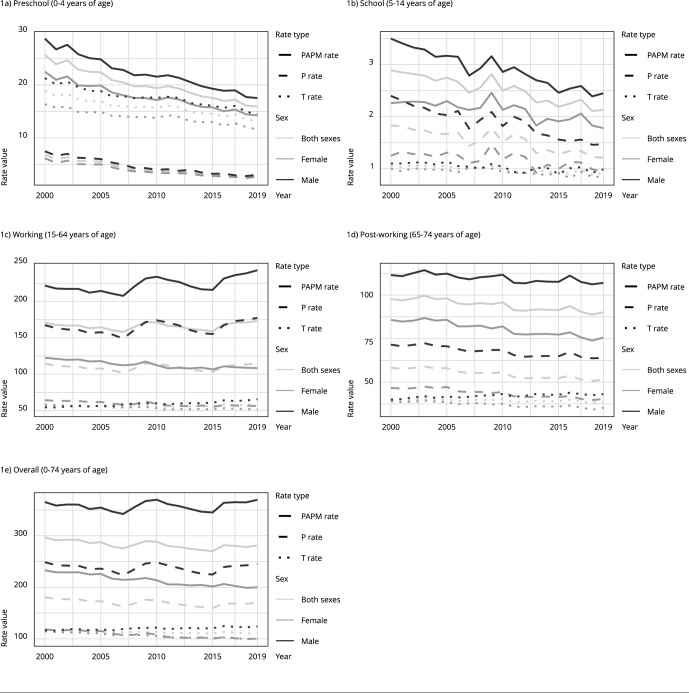
Premature and avoidable mortality rate per 100,000 deaths in Mexico from 2000 to 2019 by age group and sex.

In the general trends shown in Figure 1, our study detected a subtle decline in the PAPM across all age groups for both sexes, from 297 deaths per 100,000 in 2000 to 281 deaths per 100,000 in 2019. Disaggregating this overall rate, we found that in 2019, PPPM accounted for the largest proportion of PAPM, at 170 deaths per 100,000, while the PTPM rate was 111 deaths per 100,000.

PAPM: potentially avoidable premature mortality.

Upon closer examination, we found significant discrepancies in PAPM rates between males and females. In 2019, the PAPM rate was significantly higher in males (370 deaths per 100,000) than in females (200 deaths per 100,000). This sex disparity was further highlighted by a careful review of the different age groups. Among these, the working-age group had the highest rate (173 deaths per 100,000), followed by the post-working-age group (90 deaths per 100,000), the preschool-age group (16 deaths per 100,000), and the school-age group (2 deaths per 100,000).

When comparing preventable and treatable deaths, PPPM rates consistently exceeded PTPM rates in most age groups, including the post-working-age group (51 vs. 38 deaths per 100,000), the working-age group (115 vs. 50 deaths per 100,000), and the school-age group (1.2 vs. 0.9 deaths per 100,000). Interestingly, the preschool-age group showed a reverse pattern, with a PPPM rate of 13 deaths per 100,000 compared with a PTPM rate of 2.06 deaths per 100,000.

By analyzing trends from 2000 to 2019, Figure 1 shows the national rates by type (PPPM, PTPM, and PAPM) and age group (preschool age, school age, working age, post-working age, and overall). Most PAPM is concentrated in the working-age and post-working age groups, and the proportion is higher for PPPM, except in the preschool-age group, for which PTPM is consistently higher than PPPM.

Most age groups show a downward trend, except for the working-age group for males. This becomes clearer when observing the AAPC obtained from the joinpoint analysis. Table 1 shows the evolution of PPPM, PTPM, and PAPM over each quinquennium from 2000 to 2019 for each sex and age group. The analysis for both sexes and for all age groups over the period reveals a statistically significant average annual decrease, with an AAPC of 0.31% for PPPM, 0.02% for PTPM, and 0.28% for PAPM.

**Table 1:** Trends in premature avoidable mortality rates by period, rate type, age group, and sex from 2000 to 2019.

	Sex/Period									
	Male					Female				
	2000-2004	2005-2009	2010-2014	2015-2019	AAPC	2000-2004	2005-2009	2010-2014	2015-2019	AAPC
					2000-2019					2000-2019
Preschool age (0-4 years)										
PPPM	33.4	24.8	19.2	15.8	-	27.1	20.6	16.2	13.2	-
AAPC	-4.1 *	-7.6 *	-4.3 *	-3.0 *	-4.7 *	-3.8 *	-7.2 *	-3.9 *	-2.2	-4.2 *
PTPM	100.43	89.7	85.8	77.2	-	77.7	71.0	68.1	61.6	-
AAPC	-2.53 *	-1.3 *	-1.5 *	-2.8 *	-2.0 *	-2.0 *	-1.4 *	-1.4 *	-2.6 *	-1.7 *
PAPM	133.9	114.5	105	92.4	-	104.8	91.6	84.2	74.8	-
AAPC	-2.9 *	-2.7 *	-2.1 *	-2.7 *	-2.5 *	-2.6 *	-2.7 *	-1.9 *	-2.5 *	-2.3 *
School age (5-14 years)										
PPPM	11.1	9.9	9.1	7.6	-	6.4	6.3	5.7	5.2	-
AAPC	-3.2 *	-1.1	-4.9 *	-1.9	-2.6 *	-0.2	-0.2	-5.6 *	-2.7	-1.7 *
PTPM	5.5	5.3	4.9	4.8	-	5.0	5.0	4.6	4.4	-
AAPC	-0.7	-0.7	-1.2	-0.4	-0.8 *	-0.5	0.5	-1.5 *	-0.9	-0.8 *
PAPM	16.7	15.2	14	12.4	-	11.3	11.3	10.3	9.5	-
AAPC	-2.4 *	-1.3	-2.9 *	-0.7	-1.9 *	-0.3	-0.3	-4.1 *	-2.2	-1.4 *
Working age (15-64 years)										
PPPM	809.0	794.3	828.5	848.3	-	315.8	300.7	285.4	282.5	-
AAPC	-1.3 *	1.9 *	-3.2 *	2.9 *	0.4 *	-1.4 *	0.03	-1.2 *	0.2	-0.7 *
PTPM	276.6	291.6	298.7	317.8	-	285.1	274.1	260.8	261.7	-
AAPC	0.7	1.7 *	0.4	1.5 *	1.0 *	-0.8	-0.8 *	-0.5	-0.2	-0.6 *
PAPM	1,085.6	1,085.9	1,127.2	1,166.2	-	600.9	574.8	546.2	544.2	-
AAPC	-0.7 *	1.7 *	-2.3 *	2.6 *	0.6 *	-1.2	-0.2	-0.4 *	0.2	-0.6 *
Post-working age (65-74 years)										
PPPM	356.8	343.4	328.4	324.0	-	233.6	224.1	210.8	203.6	-
AAPC	-0.0	-1.0 *	-0.6	-0.9	-0.6 *	0.25	-1.5 *	-0.8	-1.1	-0.8 *
PTPM	204.5	209.3	212.9	215.4	-	194.6	188.7	181.3	177.2	-
AAPC	1.0 *	0.3	0.4	-0.2	0.4 *	0.03	-0.9 *	-0.5	-1.0 *	-0.6 *
PAPM	561.3	552.7	541.2	539.5	-	428.2	413.0	392.1	380.9	-
AAPC	0.3	-0.5	-0.2	-0.6	-0.2	0.1	-1.2 *	-0.7	-1.02 *	-0.7 *
Overall (0-74 years)										
PPPM	1,210.4	1172.4	1,185.2	1,195.1	-	582.8	551.7	518.1	504.5	-
AAPC	-1.3 *	1.0 *	-2.5 *	1.9 *	-0.0	-1.2	-0.7	-1.21 *	-0.3	-0.9 *
PTPM	587.1	595.9	602.3	615.2	-	562.3	538.9	514.8	504.9	-
AAPC	0.1	0.8 *	0.0	0.5	0.3 *	-0.8 *	-0.8 *	-0.64 *	-0.7 *	-0.7 *
PAPM	1,797.4	1,768.3	1,787.4	1,810.4	-	1,145.1	1,090.6	1,032.9	1,009.4	-
AAPC	-0.8 *	0.8 *	-1.7 *	1.5 *	0.1	-0.9	-0.9 *	-0.8 *	-0.6	-0.8 *

AAPC: average annual percent change; PAPM: potentially avoidable premature mortality; PPPM: potentially preventable premature mortality; PTPM: potentially treatable premature mortality.

Note: rates are shown as deaths per 100,000.

* Indicates that the AAPC is significantly different form zero at the α = 0.05 level.

However, our analysis by quinquennium shows that the AAPCs are not constant. The AAPC in PAPM from 2015 to 2019 shows a positive trend of 0.5%, mainly driven by PPPM, with an AAPC of 1.25%.

Despite the overall downward trend, the decrease is much more pronounced in the preschool age and school age groups (2.43% and -1.6% AAPC), while it is nearly flat in the workingage group. Notably, when looking at the quinquennium, there is a rapid increase of 1.82% in the AAPC from 2015 to 2019.

### Subnational analysis

The subnational analysis shows significant differences within each state, with variations by rate type and age group. This suggests that national trends may sometimes mask these disparities.

**Figure 2:**
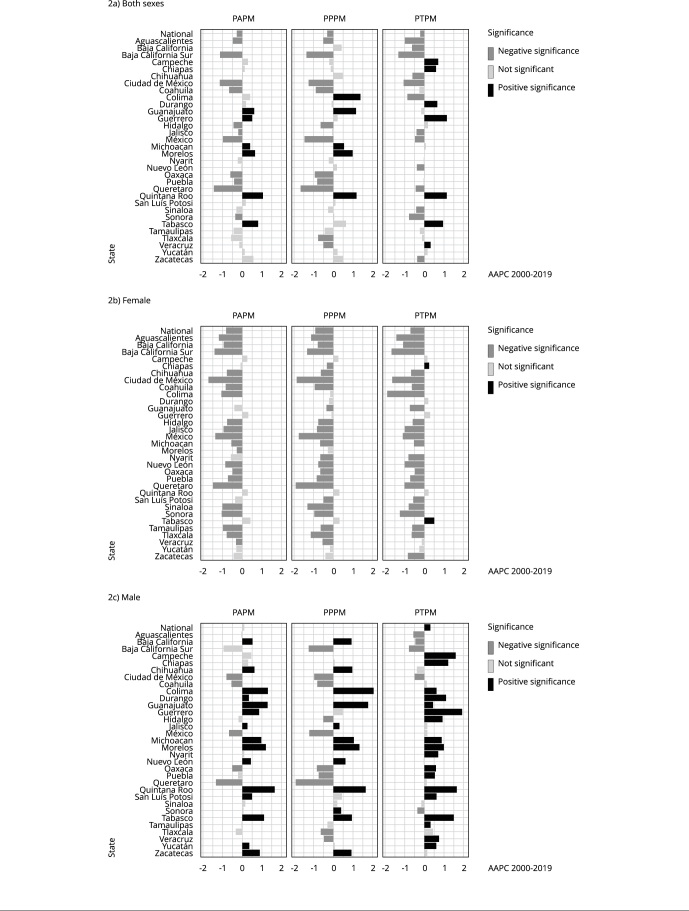
Average annual percent changes (AAPC) in potentially avoidable premature mortality (PAPM); potentially preventable premature mortality (PPPM); potentially treatable premature mortality (PTPM) by sex for overall age (0-74 years) group from 2000 to 2019 across Mexican states.

On the one hand, states such as Mexico City, Coahuila, State of Mexico, and Queretaro follow the national trend for both sexes for all ages, as shown in Figure 2, with downward trends in total PAPM for both PPPM and PTPM. Conversely, states such as Quintana Roo and Tabasco show increasing rates in all age groups.

It is crucial to highlight that PAMP component trends are not uniform across states. For instance, Chiapas and Guerrero show significant upward trends only in PTPM, while Guanajuato, Michoacan, Morelos, and Zacatecas show a significant upward trend only in PPPM. Interestingly, Colima shows diverging rates: in this state, from 2000 to 2019, PPPM rates increased while PTPM rates decreased.

A close analysis of the disparity in trends by sex shows that female rates generally show a downward trend, with the exception of PTPM in Chiapas and Tabasco. On the other hand, male trends are predominantly upward, especially in PPPM. However, there are exceptions, with downward trends in both PPPM and PTPM observed in states such as Baja California Sur and Mexico City. Other states, including Coahuila, State of Mexico, Nuevo Leon, Puebla, Queretaro, and Tlaxcala, show a decrease in PPPM only. In contrast, states such as Baja California, Hidalgo, Sonora, and Veracruz show contradictory trends, with PPPM rates increasing and PTPM rates decreasing.

**Figure 3:**
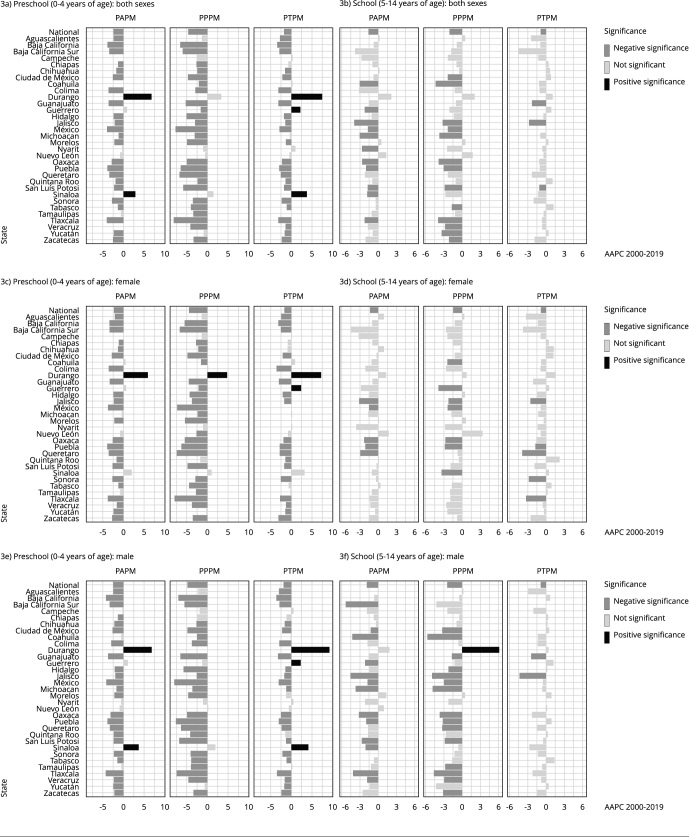
Average annual percent changes (AAPC) in potentially avoidable premature mortality (PAPM); potentially preventable premature mortality (PPPM); potentially treatable premature mortality (PTPM) by sex for the preschool age (0-4 yeares) and school age (5-14 years) groups from 2000 to 2019 across Mexican states.

**Figure 4:**
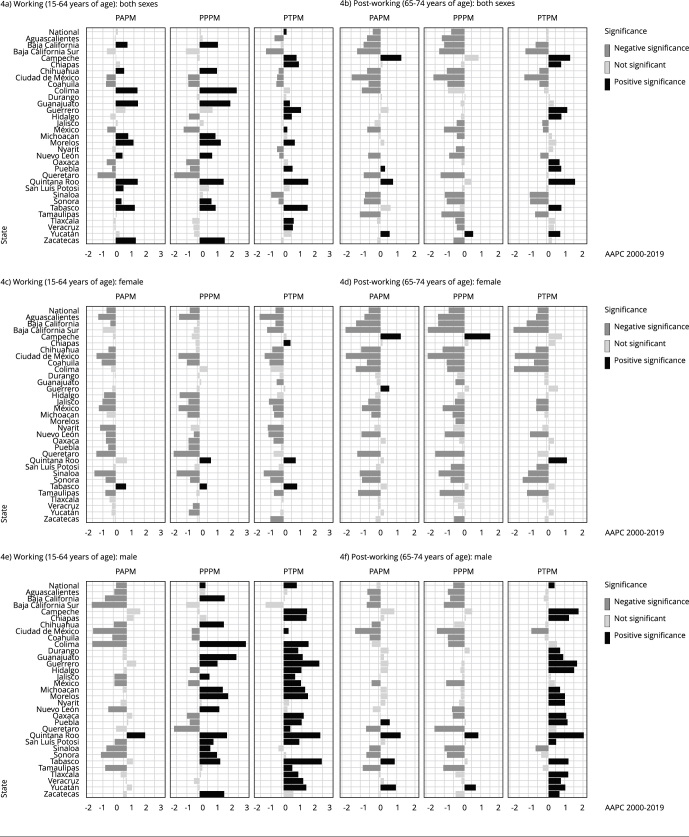
Average annual percent changes (AAPC) in potentially avoidable premature mortality (PAPM); potentially preventable premature mortality (PPPM); potentially treatable premature mortality (PTPM) by sex for the working age (15-64 years) and post-working age (65-74 years) groups from 2000 to 2019 across Mexican states.

Analysis by age group, as shown in Figures 3 and 4, reveals that sex differences are rare in the preschool age and school age groups. The general trends show a decrease in rates, particularly PPPM, especially in the preschool age group. Notably, some states, such as Durango and Sinaloa, show increasing trends for the preschool age group. Durango, in particular, has a rising PAPM rate, with an average annual increase of 6.8% over the period studied, largely attributed to preventable deaths. In the school age group, Durango’s PPPM trend for males stands out, as it is the only state that shows a significant upward trend, with an average annual increase of 6%.

When analyzing the working age and post working-age groups (Figure 4), clearly conflicting trends between sexes can be observed. For the working age group, the majority of states show decreasing rates for females, with the exceptions of Chiapas, Quintana Roo, and Tabasco. For men, most states show upward trends, with some exceptions, such as Coahuila. Mexico City, Hidalgo, State of Mexico, Oaxaca, Puebla, and Queretaro show contradictory trends, with a decrease in PPPM and a rise in PTPM rates.

In the post-working age group, the pattern largely mirrors that of the working age group, characterized by generally downward trends in females and upward trends in males. A notable difference is that the increasing trends for males are predominantly concentrated in PTPM, while the decreasing trends for females the exceptions in Campeche, Guerrero, and Quintana Roo, which have rising trends. In Mexico City and Sinaloa, significantly decreasing overall PAMP rates and components stand out among males. Other positive outliers for males include Aguascalientes, Baja California, Baja California Sur, Chihuahua, Colima, Coahuila, State of Mexico, Nuevo Leon, Oaxaca, Puebla, Queretaro, Sonora, and Tamaulipas, all of which have decreasing PPPM rates -even when PTPM increases or does not significantly decrease.

## Discussion

Our study provides insight into the dynamics of PAPM in Mexico from 2000 to 2019. We observed a slight decrease in national PAPM rates, from 297 deaths per 100,000 in 2000 to 281 deaths per 100,000 in 2019. Notably, PPPM rates (170 deaths per 100,000) significantly exceeded PTPM rates (111 deaths per 100,000). The disparity between these rates became increasingly pronounced in 2019, particularly among males and females in the working-age group. These patterns are consistent with findings from the PAHO ^7^.

During the study period, most age groups showed a decrease in PAPM rates. An exception was the upward trend among working-age males, which is consistent with global data showing an overall decline in PAPM, although patterns vary between countries ^27^ ^,^ ^28^. Our data are in line with PAHO findings, showing a general decline in PAPM over recent decades. However, the alarming occurrence of approximately 2.5 million premature deaths in 2019, due to chronic diseases and external factors, overshadows this positive trend. The 12.3% disparity in PAPM rates between Mexico and the Americas in 2019 is a notable example. The upward trend in PAPM in Mexico, particularly among males, may be linked to an increase in violence-induced deaths, as detailed by Gutiérrez-Reyes et al. ^19^.

A detailed examination of state-specific trends revealed significant deviations from the overall national trend. While some states, such as Mexico City, Coahuila, and State of Mexico, followed the national downward trend, others, such as Quintana Roo and Tabasco, experienced concerning increases in mortality rates. The complexity becomes even more apparent when analyzing PAPM components: for example, Chiapas and Guerrero saw a rise exclusively in PTPM, while Guanajuato and Michoacan experienced an increase solely in PPPM. These complex trends, further segmented by age, indicate a multifaceted interplay of regional and demographic determinants, aligning with global observations such as the pronounced disparities in remote Canadian communities and the variance in under-five mortality rates in Colombia ^10^ ^,^ ^11^.

This study also drew attention to the social determinants of health, such as education and access to health care, and their significant impact on health and well-being ^29^. The observed disparities in PAPM rates across states likely stem from variations in these social determinants, reflecting underlying socioeconomic differences. This highlights the influence of state-level policies and health care management on mortality variation and highlights the need for tailored interventions to effectively address these disparities. Further research is needed to explore the impact of these determinants and ensure that strategies are both well-informed and effectively implemented.

In addition, the study highlighted the notably higher PAPM rates among males and within the working-age population, predominantly due to preventable diseases. This trend, however, varied between groups, with men in the working-age group experiencing an upward trend, which may be linked to the increasing number of violent deaths in the country over the past decades ^19^.

The use of PAPM as a health outcome measure has limitations, including the inability to distinguish between chronic and acute diseases, which may underestimate the true impact of illness. In the preschool-age group, the accuracy of mortality data can be compromised by factors such as underreporting or misclassification of cause of death ^30^. Therefore, it is crucial to complement PAPM with other measures for a comprehensive health assessment, especially in younger children, for whom data issues may be more pronounced.

Our findings suggest that socioeconomic factors significantly influence health outcomes. Lower socioeconomic status often correlates with higher rates of preventable disease and limited access to health care, contributing to increased PAPM rates ^17^. The observed variation in PAPM rates across states and between different demographic groups within states likely reflects these underlying socioeconomic differences. Space constraints limited our ability to conduct a more in-depth analysis of regional disparities. Nevertheless, this highlights the need for specific, regionally focused analyses to better understand and address this variation.

The marked variability in PAPM trends, particularly in PPPM and PTPM across different states, underscores the complexity of health disparity dynamics within a nation. While some states follow national trends, others deviate significantly, indicating that complex local factors influence these outcomes. The different behaviors of PAMP components in states such as Chiapas, Guerrero, Guanajuato, Michoacan, Morelos, and Zacatecas suggest that the impact of interventions may vary depending on the specific area and target population within a state. In addition, the divergent trends in Colima highlight the fact that different aspects of public health can progress in opposing directions.

Age-based disparities in mortality rates add to this complexity. The consistent disparities observed in the working-age group contrast sharply with the fluctuating trends in the school-age and preschool-age groups, indicating the need for age-specific interventions.

Differences in avoidable mortality by sex, age group, and federal unit are well documented and have been previously described in the literature ^31^ ^,^ ^32^. However, the significance of our study is that it highlights the persistence of these differences and, in some cases, the widening of these gaps over time. This paper underscores the enduring nature of these disparities and adds to the existing body of evidence by demonstrating their persistence and, in some cases, exacerbation, thus emphasizing the continued importance of addressing and mitigating avoidable mortality via targeted public health interventions and policies.

In conclusion, our study provides valuable insights into the heterogeneous landscape of PAPM rates across different age groups, sexes, and states within the nation. Notably, a significant proportion of PAPM rates are attributable to the working-age group, particularly among males. By highlighting these variations in trends, we underscore the need for further research aimed at designing and implementing more tailored, locally focused health interventions in the future.

Crucially, future research should aim to establish which social determinants of health influence these outcomes and identify the socioeconomic factors that disproportionately affect males and females. While PAPM provides informative measures, it is essential to acknowledge its limitations and complement it with other health outcome metrics for a comprehensive understanding of the health status of different groups. Given that these disparities are likely driven by variations in social determinants of health and socioeconomic factors, future work should prioritize identifying and addressing these specific contributors.

## References

[1] Organization for Economic Co-operation and Development (2019). Health at a glance 2019: OECD indicators.

[2] Organización Panamericana de la Salud (2024). Salud en las Américas. Mortalidad prematura potencialmente evitable.

[3] Organization for Economic Co-operation and Development (2021). Health at a glance 2021: OECD indicators. Avoidable mortality (preventable and treatable).

[4] Castelli A, Nizalova O (2011). Avoidable mortality: what it means and how it is measured..

[5] Office for National Statistics (2012). Definition of avoidable mortality.

[6] Allin S, Veillard J, Wang L, Grignon M (2015). How can health system efficiency be improved in Canada. Healthc Policy.

[7] Pan American Health Organization Potentially avoidable premature mortality: what is it and why is it relevant?.

[8] Gulliford M, Figueroa-Munoz J, Morgan M, Hughes D, Gibson B, Beech R (2002). What does "access to health care" mean. J Health Serv Res Policy.

[9] UCL Institute of Health Equity (2014). Local action on health inequalities: introduction to a series of evidence papers.

[10] Gómez-Dantés O, Flamand L, Cerecero-García D, Morales-Vazquez M, Serván-Mori E (2023). Origin, impacts, and potential solutions to the fragmentation of the Mexican health system a consultation with key actors. Health Res Policy Syst.

[11] Moreno-Jaimes C, Flamand L (2016). Towards health-care equality? The performance of Seguro Popular in México (2003-2013).. Journal of Public Governance and Policy: Latin American Review.

[12] Gutiérrez JP, García-Saisó S, Dolci GF, Ávila MH (2022). Effective access to health care in Mexico. BMC Health Serv Res.

[13] Observatorio Nacional de Inequidades en Salud (2019). Primer informe sobre desigualdades en salud en México..

[14] Gutierrez JP, Agudelo-Botero M, Garcia-Saiso S, Zepeda-Tena C, Davila-Cervantes CA, Gonzalez-Robledo MC (2020). Advances and challenges on the path toward the SDGs subnational inequalities in Mexico, 1990-2017. BMJ Glob Health.

[15] González Guzmán R (2018). Inequidades en salud en México 2015-2016. Salud Probl.

[16] Gómez-Dantés H, Fullman N, Lamadrid-Figueroa H, Cahuana-Hurtado L, Darney B, Avila-Burgos L (2016). Dissonant health transition in the states of Mexico, 1990-2013: a systematic analysis for the Global Burden of Disease Study 2013.. Lancet.

[17] Lewer D, Jayatunga W, Aldridge RW, Edge C, Marmot M, Story A (2020). Premature mortality attributable to socioeconomic inequality in England between 2003 and 2018: an observational study.. Lancet Public Health.

[18] Abbafati C, Abbas KM, Abbasi-Kangevari M, Abd-Allah F, Abdelalim A, Abdollahi M (2020). Global burden of 369 diseases and injuries in 204 countries and territories, 1990-2019: a systematic analysis for the Global Burden of Disease Study 2019.. Lancet.

[19] Gutiérrez-Reyes JP, Calderón OV, Prado AC (2023). Tendencia de las inequidades en homicidios en México para el periodo de 2000 a 2021: análisis ecológico longitudinal.. Rev Panam Salud Pública.

[20] Aburto JM, Beltrán-Sánchez H, García-Guerrero VM, Canudas-Romo V (2016). Homicides in Mexico reversed life expectancy gains for men and slowed them for women, 2000-10. Health Aff (Millwood).

[21] Organization for Economic Co-operation and Development (2015). Measuring well-being in Mexican states.

[22] Organization for Economic Co-operation and Development (2023). Working age population (indicator).

[23] Gobierno de México Proyecciones de la población de México y de las Entidades Federativas, 2016-2050..

[24] Instituto Nacional de Estadística y Geografía Estadísticas de defunciones registradas (EDR). Mortalidad..

[25] Organisation for Economic Co-operation and Development Avoidable mortality: OECD/Eurostat lists of preventable and treatable causes of death (January 2022 version)..

[26] Ahmad OB, Boschi-Pinto C, Lopez AD, Murray CJL, Lozano R, Inoue M (2001). Age standardization of rates: a new WHO standard..

[27] Martinez R, Lloyd-Sherlock P, Soliz P, Ebrahim S, Vega E, Ordunez P (2020). Trends in premature avertable mortality from non-communicable diseases for 195 countries and territories, 1990-2017: a population-based study.. Lancet Global Health.

[28] Pérez Contreras M, Márquez Calderón S (2021). Mortalidad prematura potencialmente evitable en Andalucía, 2000-2019: análisis de tendencias.

[29] Organización Panamericana de la Salud (2018). Sociedades justas: equidad en la salud y vida digna. Resumen Ejecutivo del Informe de la Comisión de la Organización Panamericana de la Salud sobre Equidad y Desigualdades en Salud en las Américas.

[30] Hernandez B, Rodriguez Angulo E, Johnson LM, Palmisano EB, Ojeda R, Ojeda R (2022). Assessment of the quality of the vital registration system for under-5 mortality in Yucatan, Mexico. Popul Health Metr.

[31] Dávila-Cervantes CA, Agudelo-Botero M (2015). Mortalidad evitable en México y su contribución a los años de vida perdidos Análisis por grado de marginación estatal, 2001-2010. Papeles de Población.

[32] Hernández HG, Cervantes CAD (2022). Amenable mortality analysis in Mexico during the period 1998-2019. Poblac Salud Mesoam.

